# Tumor-targeted *in vivo* gene silencing *via* systemic delivery of cRGD-conjugated siRNA

**DOI:** 10.1093/nar/gku831

**Published:** 2014-09-15

**Authors:** Xiaoxia Liu, Wei Wang, Dmitry Samarsky, Li Liu, Qian Xu, Wenqing Zhang, Guangzu Zhu, Ping Wu, Xialin Zuo, Houliang Deng, Jingjing Zhang, Zhuomin Wu, Xiaohui Chen, Lingfeng Zhao, Zhiyong Qiu, Zhongyi Zhang, Qiyi Zeng, Wei Yang, Biliang Zhang, Aimin Ji

**Affiliations:** 1 Department of Pharmacy, Zhujiang Hospital, Southern Medical University, Guangzhou 510282, China; 2 Guangzhou RiboBio Co., Guangzhou 510663, China; 3 Department of Cell Biology, Southern Medical University, Guangzhou 510515, China; 4 Guangdong Shuanglin Bio-pharmaceutical Co.,Ltd, Zhanjiang 524005, China; 5 Witspool Biopharmaceutical Co., Wuhan 430079, China; 6 Department of Pediatrics, Zhujiang Hospital, Southern Medical University, Guangzhou 510282, China; 7 Center for Drug Non-clinical Evaluation and Research (National Guangzhou Key Laboratory for New Drug Safety Evaluation and Research), Guangzhou General Pharmaceutical Research Institute, Guangzhou 510240, China; 8 Guangzhou Institutes of Biomedicine and Health, Chinese Academy of Sciences, Guangzhou 510530, China

## Abstract

RNAi technology is taking strong position among the key therapeutic modalities, with dozens of siRNA-based programs entering and successfully progressing through clinical stages of drug development. To further explore potentials of RNAi technology as therapeutics, we engineered and tested VEGFR2 siRNA molecules specifically targeted to tumors through covalently conjugated cyclo(Arg-Gly-Asp-d-Phe-Lys[PEG-MAL]) (cRGD) peptide, known to bind αvβ3 integrin receptors. cRGD-siRNAs were demonstrated to specifically enter and silence targeted genes in cultured αvβ3 positive human cells (HUVEC). Microinjection of zebrafish blastocysts with VEGFR2 cRGD-siRNA resulted in specific inhibition of blood vessel growth. In tumor-bearing mice, intravenously injected cRGD-siRNA molecules generated no innate immune response and bio-distributed to tumor tissues. Continuous systemic delivery of two different VEGFR2 cRGD-siRNAs resulted in down-regulation of corresponding mRNA (55 and 45%) and protein (65 and 45%) in tumors, as well as in overall reduction of tumor volume (90 and 70%). These findings demonstrate strong potential of cRGD-siRNA molecules as anti-tumor therapy.

## INTRODUCTION

RNAi gene silencing technology, with siRNAs as its triggers, is based on natural intracellular mechanisms and has a strong potential as novel therapeutic strategy for a broad range of diseases—from genetic disorders to cancer and viral infection (1,2). To become widely applied in the clinic this technology has to address several challenges, including stability of siRNA molecules *in vivo*, delivery and bio-distribution to intended tissues/organs, as well as potential non-specific (e.g. off-target and immunostimulatory) effects. While stability and specificity of siRNAs *in vivo* has been improved using various chemical modifications (2), *in vivo* delivery remains the biggest difficulty, due to the large size (∼13 kDa) and strong relative negative charge of the molecules. Numerous approaches have been explored with varying success, including assembly of siRNAs into nanoparticles with cationic polymers (3) and lipids (4), integration into exosomes (5), complexation with peptide containing antibodies and nucleic acid binding domains (6), as well as siRNA conjugation with peptide transduction domains (7), cell-specific aptamers (8), receptor-specific ligands, such as cholesterol (9), alpha-tocopherol (10), lauric acid (11), receptor-specific agonist (12) and cell growth factor (or its peptide analogue) (13).

Integrin αvβ3 plays an important role in angiogenesis and tumor metastasis, and is significantly up-regulated in tumor blood vessels, as well as in invasive tumor cells of many cancer types (but not in cells of quiescent endothelium and normal tissues) (14). High affinity of cancer-related integrin αvβ3 to arginine–glycine–aspartate (RGD) peptide has prompted the use of RGD as ligand for tumor targeting liposomes (15). In a recent *in vitro* study, siRNA conjugated to cyclic RGD (cRGD) was also demonstrated to selectively enter cells expressing αvβ3 integrin (16). We explored further the potential of cRGD-conjugated siRNAs through additional cell culture and whole animal studies.

As a test target for the new cRGD-siRNA constructs, we chose VEGFR2 gene. Vascular endothelial growth factors (VEGFs) and corresponding receptors (VEGFRs) participate in the regulation of blood vessel development from precursor cells during early embryogenesis, as well as in the formation of new blood vessels from pre-existing vessels at later stages (17). In solid tumors VEGF is mainly produced by cancer cells, and the binding of VEGF to VEGFR2 activates multiple cellular pathways important for tumor angiogenesis (18). Therefore, effective delivery of VEGFR2 cRGD-siRNAs was expected to inhibit angiogenesis and, consequently, progression of tumor growth *in vivo*.

## MATERIALS AND METHODS

### Human cell culturing

HUVEC (human umbilical vein endothelial cells) were purchased from ATCC (CRL-1730). A549-luc (luciferase expressing) cells were kindly provided by RiboBio Co., Ltd (Guangzhou, China). HeLa cells were kindly provided by Department of Hematology, Zhujiang Hospital, Southern Medical University (Guangzhou, China). Cells were grown and cultured using supplier recommended reagents and media according to standard protocols and procedures.

### Preparation of cRGD- and cRAD-conjugated siRNA molecules

The test cyclo(Arg-Gly-Asp-d-Phe-Lys[PEG-MAL]) (cRGD) and the control cyclo(Arg-Ala-Asp-D-Phe-Lys[PEG-MAL]) (cRAD) peptides, where PEG stands for 8-amino-3,6-dioxaoctanoic acid and MAL—for β-maleimidopropionic acid, were synthesized by Peptide International (Kentucky, USA). Commercially available 5′-O-(4,4′-dimethoxytrityl)-2′-O-t-butyl-dimethyl-silyl-3′-O-(2-cyanoethyl-N,N-diisopropyl) RNA and corresponding 2′-deoxy-thymidine and 2′-O-methyl phosphoramidite monomers of 6-N-benzoyladenosine (ABz), 4-N-acetylcytidine (CAc), 2-N-isobutyrylguanosine (GiBu) and uridine (U) (Sigma-Aldrich, USA) were used for unmodified and 2′-O-methyl sugar modified RNA synthesis. The 3′-end C6 thiol modifications were introduced to RNA molecules using commercially available thiol-modifier C6 S-S phosphoramidite (Glen Research, USA). Passenger (sense) and guide (antisense) strands of siRNAs were individually synthesized and purified according to standard oligonucleotide synthesis and deprotection protocols (19). The Cy5 phosphoramidites (Guangzhou RiboBio Co., Ltd Supplementary Figure S1) were used to attach the fluorescent label to the 5′-ends of siRNA antisense strands.

The peptide-siRNA conjugation was performed as follows. 100 nmol of 3′-thiol-modified sense RNA strand was dissolved in 950 μl of 100 mM HEPES-KOH buffer (pH 7.2). Subsequently, 500 nmol of cRGD or cRAD in 50 μl water was added. Resulting solution was saturated with N_2_ to remove traces of air, and the Michael reaction was carried out at 4°C. Efficiency of the reaction was tested using 20% denaturing urea-polyacrylamide gel electrophoresis, and quantitative conversion into conjugated RNA was confirmed after 16 h. Resulting solution was centrifuge filtered (MW ≤ 3000), washed 3× with 10 ml RNase free water to remove excess of cRGD or cRAD, and then freeze-dried to yield the cRGD-ssRNA or cRAD-ssRNA (single stranded) conjugates. Final products were detected and characterized using LC-MS. To generate double-stranded siRNAs, antisense RNA molecules (50 μM) were mixed with cRGD-ssRNA or cRAD-ssRNA conjugates (50 μM) in annealing buffer (10 mM Tris-HCl pH 7.4, 50 mM NaCl, and 1 mM ethylenediaminetetraacetic acid), heat denatured at 95°C for 3 min and slowly cooled down to 20°C. Desalting and filter purification of siRNAs for *in vivo* applications were performed using Millipore centrifugation with 0.22 μm sterile filtration membrane.

### Serum stability

Five microliter of 20 μM cRGD-siRNA was mixed with 5 μl of mouse serum and incubated at 37°C for 0, 24, 48 and 72 h. Aliquots were taken at each of the time points and subjected to electrophoresis in 1.2% non-denaturing agarose gels.

### Flow cytometry

0.1 ml of cell suspension (HeLa or HUVEC cells; 1 × 10^6^ cells/ml) was mixed with 5 μl (1 μg per test) primary monoclonal anti-integrin αvβ3 antibodies (eBioscience; Catalog No 11-0519) at 37°C for 30 min. After that procedure, cells were collected, washed twice with phosphate buffered saline (PBS) and resuspended in 0.1 ml PBS. Fluorescence data were collected using FACSCalibur cell sorting system (BD Biosciences, USA) and analyzed using CellQuest 3.0 software.

### Cytotoxicity analysis in cell culture

Cells were transfected with 100 nM (final concentration) cRGD-siRNA or non-conjugated siRNA using Lipofectamine 2000 (Life Technologies, USA) according to manufacturer's protocols. Following 24-h incubation at 37°C, 10 μl of CCK-8 (Beyotime, China) solution was added to each well. Plates were incubated at 37°C for additional 1 h and optical densities were recorded at 450 nm using a Microplate reader (Bio-Rad, USA). Cell viability was plotted as a percentage of untreated control cells.

### Confocal laser scanning microscopy

HUVEC cells were transfected with 100 nM of Cy5-labeled cRGD-siRNA or Cy5-labeled cRAD-siRNA in Dulbecco's modified Eagle's medium (DMEM) containing 10% fetal bovine serum (FBS) and HeLa cells were transfected with 100 nM of Cy5-labeled cRGD-siRNA in DMEM containing 10% FBS. Alternatively, HUVEC cells were incubated with primary monoclonal anti-integrin αvβ3 antibodies (10 μl, 1 mg/ml; PL laboratories) or with cRGD (cRAD) peptide (20 μl, 1 mg/ml) at 37°C for 30 min, and subsequently transfected with 100 nM of Cy5-labeled cRGD-siRNA. Twelve hours after transfection cells were washed with chilled PBS and fixed immediately using histiocyte stationary liquid (4% paraformaldehyde) at room temperature for 10 min, followed by nuclei staining with 4,6-diamidino-2-phenylindole (DAPI) (Roche, Switzerland) for 10 min at 37°C. Cells were then washed 3× with PBS and used for confocal microscopy (Olympus, USA; Cy5 excitation = 640 nm, emission = 680 nm).

### qRT-PCR

For the *in vitro* tests, total RNA was extracted from cells 48 h after siRNA transfection. For the analysis of silencing duration, total RNA was extracted from cells at indicated time points after transfection. For the *in vivo* experiments, tumors were excised and total RNA was extracted using Trizol reagent (Life Technologies, USA), according to manufacturer's protocol. For the cDNA synthesis, 1 μg of extracted RNA was reverse transcribed using ReverTra Ace-α-^®^ cDNA Synthesis Kit (Toyobo, Japan). Sequences of the primers used for reactions are listed in Supplementary Table S1. Real-time monitoring of PCR amplification was conducted using the Applied Biosystems 7500 Real-Time PCR System (Life Technologies, USA) and the SYBR Green Realtime Master Mix (Toyobo, Japan).

### Western blot analysis

Western blot analyses to detect VEGFR2 protein levels were performed as described previously (20). The bands were scanned using Epson Perfection V370 Photo Scanner (Epson, Japan). The band intensities for VEGFR2 were normalized to those of glyceraldehyde-3-phosphate dehydrogenase (GAPDH) or β-actin, and calculated using ImageJ software (National Institutes of Health, USA).

### Angiogenesis assay in zebrafish

Wild-type and *Tg(flk1:EGFP)* zebrafish lines were used for *in vivo* model systems. Both were maintained as described in the zebrafish handbook. 0.003% phenylthiourea (PTU) (Sigma, USA) was used to block pigment formation in cultured embryos.

Zebrafish embryos that looked healthy and well developed were collected 5 hours post-fertilization (hpf), using dissecting microscope and 20 embryos/well were plated into the 6-well plates. cRGD-siVEGFR2 (zebrafish) (100 μM, 2 nl), siVEGFR2 (zebrafish) (100 μM, 2 nl), cRGD (2 mg/ml, 2 nl) or ddH_2_O (2 nl) were microinjected into the 5 hpf embryos. Blood-vessels were detected using stereo fluorescence microscopy (Olympus, USA) after incubation of embryos at 28.5°C for 24 h and using confocal microscopy (Olympus, USA) after 72 h incubation.

### Animal handling

All animal experiments were conducted at the Zhongshan School of Medicine,Sun Yat-sen University (Guangzhou, China), according to protocols and procedures authorized by the hosting institution.

### Tumor biology

BALB/c nude mice (female; 4–6 weeks; ∼20 g) were injected subcutaneously on the right side of the back with 5 × 10^6^ A549 luciferase-expressing cells (A549-luc). When tumor volume reached 40–50 mm^3^, animals were randomly separated into several groups for different treatments. Mice groups bearing A549-luc tumors were then treated with 150 μl intravenous injections (every 3 days; total of six injections), containing either 1 nmol VEGFR2 cRGD-siRNA1 (∼0.753 mg/kg; *n* = 10), 1 nmol VEGFR2 cRGD-siRNA2 (∼0.753 mg/kg; *n* = 5), 1 nmol VEGFR2 cRAD-siRNA2 (∼0.753 mg/kg; *n* = 5), 1 nmol cRGD alone (∼0.045 mg/kg; *n* = 5), 1 nmol β-actin cRGD-siRNA (∼0.753 mg/kg; *n* = 5), 1 nmol control cRGD-siRNA (∼0.753 mg/kg; *n* = 5) or saline (*n* = 5). Tumor diameters were measured using a caliper, and the volumes were calculated using the formula: Volume = $\frac{1}{2}$ × Length × (Width)^2^, where the ‘Length’ corresponded to the longest and the ‘Width’—to the shortest of the measured tumor diameters. The growth curves were plotted as mean tumor volumes ±SD (standard deviation). Animals were euthanized 2 days after the last treatment, tumors excised and preserved in liquid nitrogen for further analysis.

For the tissue distribution study, mice were randomly assigned to several treatment groups (*n* = 3 per each group). Then 1 nmol cRGD-siRNA (Cy5-labled), cRAD-siRNA (Cy5-labled) or non-conjugated siRNA molecules were injected intravenously at single doses. Luciferin was injected intraperitoneally at a dose of 150 mg/kg. At different time points after the injection, mice were anaesthetized by inhalation with isoflurane, following standard procedure, and *in vivo* bioluminescence imaging was conducted using Xenogen IVIS Spectrum Imaging System (PerkinElmer, USA) in an auto exposure setup (excitation 640 nm). Images were recorded using a built-in CCD camera.

### Serum chemistry analysis

For the liver toxicity studies, blood was drawn from cRGD-siRNA and saline treated animals 2 days after the last of six injections (each injection every 3 days), serum isolated, and the ALT (alanine aminotransferase) and Cr (creatinine) measurements were performed using automated Aeroset Chemistry Analyzer (Abbott, USA), following manufacturer's recommendations.

### ELISA

Athymic nude female mice (nu/nu) were injected with 1 nmol (∼0.753 mg/kg; *n* = 3) of cRGD-siRNA or 1 nmol of cRGD alone (∼0.045 mg/kg; *n* = 3) in 150 μl of saline. Mice (*n* = 3) injected with saline alone (200 μl) were used as negative controls. Six hours and 24 h after the injection, 300 μl of blood was drawn from each mouse and the serum was collected. Levels of serum cytokines IFN-α, IFN-γ, IL-6 and IL-12 were determined by ELISA, following manufacturer's recommendations (eBioscience/Affymetrix, USA).

### Immunohistochemistry and TUNEL staining

Tumor tissues were embedded in Tissue-Tek O.C.T. (optimum cutting temperature) compound medium (VWR/Sakura Finetek, USA) and frozen in dry ice powder. Frozen tumor tissues were cut into 6 μm thick sections and stained for endothelial cells using CD31, clone MEC 13.3 antibodies (BD Biosciences, USA). Appropriate biotinylated secondary antibodies, streptavidin Horseradish Peroxidase (HRP) kit and Diaminobenzidine (DAB) substrate kit were used for color development, and all the sections were counterstained with Harris hematoxylin. Tissue sections were processed for terminal dexynucleotidyl transferase(TdT)-mediated dUTP nick end labeling (TUNEL) analysis using the *In situ* Cell Death Detection Kit, POD (Roche, Switzerland) to measure apoptosis according to manufacturer's protocol.

### Statistical analysis

Where appropriate, the results are expressed as mean ±SD. *P*-values of 0.05 or less were accepted as indicators of statistically significant data. Student's *t*-tests were conducted to compare each treatment group to every other, and adjusted by ANOVA for multiple comparisons. Independent-samples *t*-tests were used to compare two groups. Each experiment was repeated 3×.

## RESULTS

### Peptide-siRNA conjugates enter cells without transfection reagent *in vitro* and produce effective, specific and sustainable gene knockdown

**Figure 1.**
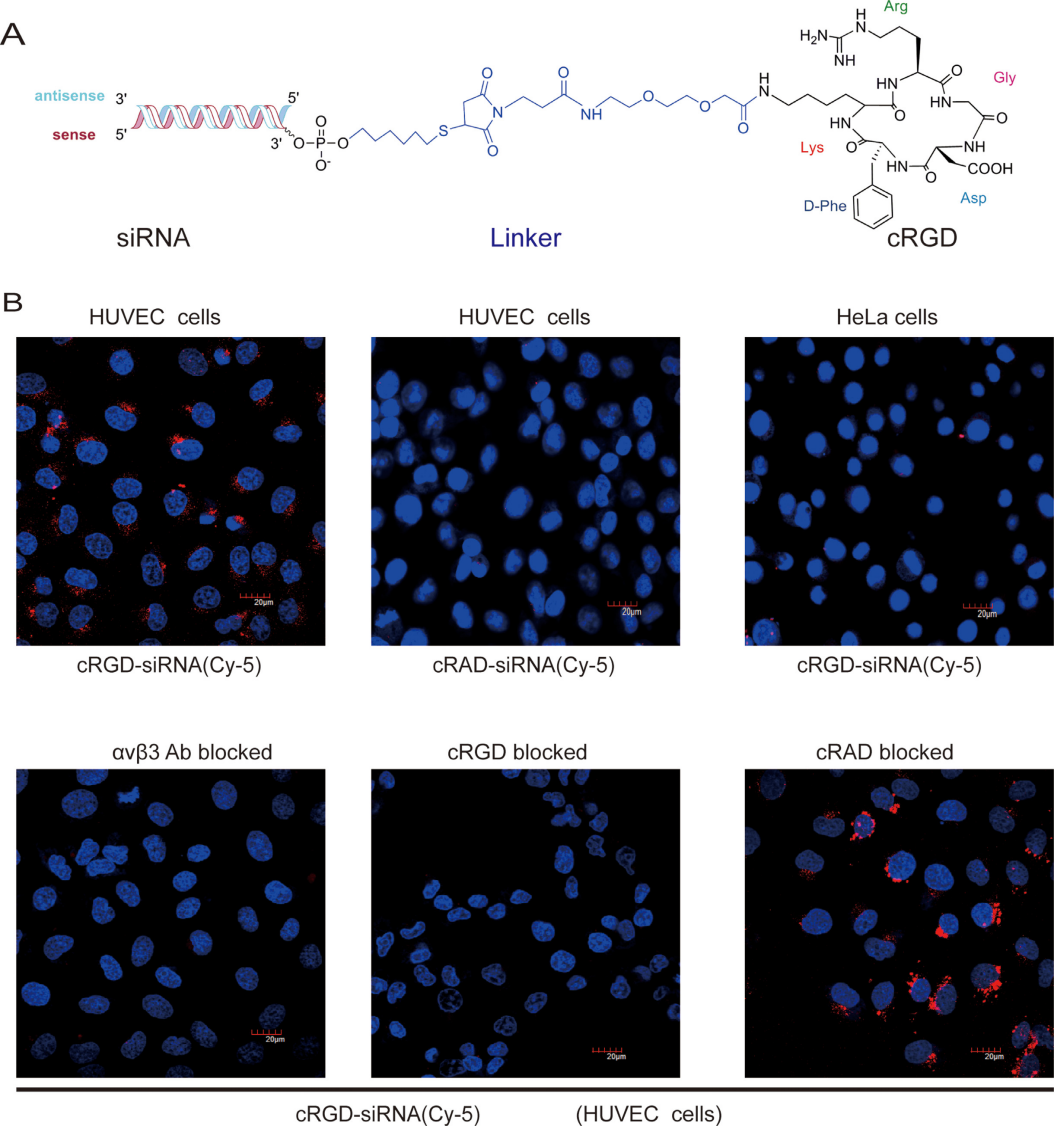
Unassisted uptake of conjugated cRGD-siRNA molecules by integrin αvβ3 expressing cells and gene silencing *in vitro*. (**A**) Schematic depiction of cRGD-siRNA conjugates. In these molecules, the cRGD moiety (right) is covalently conjugated to the 3′-phosphate of passenger (sense) strand of a gene-targeting siRNA (left) through the thiol-maleimide linker (middle). (**B**) Visualization and testing of specificity of cRGD-siRNA unassisted uptake *in vitro*. Human HUVEC (αvβ3-positive) or HeLa (αvβ3-negative) cells were treated (note: no transfection reagent was used) with Cy5-labled cRGD-siRNA or control cRAD-siRNA molecules (upper panels). For additional specificity tests, before incubation with Cy5-cRGD-siRNA HUVEC (αvβ3-positive) cells were pre-treated with αvβ3-specific antibodies, cRGD peptide, or cRAD peptide (lower panels). Twelve hours after incubation with conjugate molecules, cells were fixed and visualized using confocal laser microscopy.

To prepare peptide-siRNA (cRGD- or control cRAD-siRNA) conjugate molecules, peptides were covalently linked to the 3′-end of siRNA passenger (sense) strands using thiol-maleimide linker. Schematic diagram of a cRGD-siRNA conjugate molecule is shown in Figure 1A. An exemplary sequence/structure of the mouse VEGFR2 siRNA conjugate component is as follows. The passenger (sense) strand is 5′-mCmGmGAGAAGAAUGUGGUmUmAmAdTdT-3′ and the 5′-phosphorylated guide (antisense) strand is 5′-PmUmUmAACCACAUUCUUCUmCmCmGdTdT-3′, where ‘mN’ indicates 2′-O-methyl ribose modification (2′-O-Me) in corresponding nucleotides. Sequences for siRNAs against human, mouse and zebrafish VEGFR2 mRNAs, and their precise structures are listed in Supplementary Table S2. Control (including positive and negative) siRNAs, containing the same modifications were provided by Guangzhou RiboBio, Corp. (China). Purity of peptide-conjugated siRNAs was determined as >90% using HPLC analysis (exemplary chromatogram is shown in Supplementary Figure S1A). All siRNAs were desalted or HPLC-purified, and the molecule purity was normally higher than 80% for *in vitro* and higher than 90% for *in vivo* studies. Experimentally determined molecular weights of conjugate molecules composed of peptides and siRNA sense strands were in acceptable approximation with formula-based calculations. An exemplary case is illustrated in Supplementary Figure S1B, where a cRGD-conjugated sense strand siRNA was determined as 7977.9 Da using mass-spectrometric analysis, which was acceptably close to the predicted 7975.3 Da. Expected and observed masses of other cRGD-conjugated sense and antisense stands, as well as of Cy5-labled molecules are listed in Supplementary Table S2. The sodium dodecylsulphate-polyacrylamide gel electrophoresis analysis revealed a characteristic shift in molecular weight-dependent mobility between the conjugated and non-conjugated molecules (Supplementary Figure S1C).

Incorporation of 2′-O-Me modifications into siRNAs has been previously demonstrated to enhance molecule's resistance to RNA nucleases and to minimize potential non-specific activation of toll-like receptor-mediated immune response and toxicity (21). Consistently with this, incubation of 2′-O-Me modified cRGD-siRNA conjugates in mouse serum revealed no detectable degradation after up to 36 h and very little degradation after 48 and 72 h (Supplementary Figure S1D). At similar conditions, unmodified RNA oligonucleotides are typically degraded within less than an hour (data not shown).

It has been previously demonstrated that HUVEC express integrin αvβ3 on their cell surface (22), while human HeLa cells are avβ3 negative (23). This earlier observation was confirmed by our cell sorting experiment using αvβ3 antibodies (Supplementary Figure S2). Based on this, HUVEC and HeLa cells were selected to explore efficiency and specificity of cRGD-siRNA performance in the following *in vitro* studies.

For visualization of cellular uptake, the test cRGD-siRNA molecules were labeled with fluorophore (Cy5), and fluorescence patterns were monitored using confocal laser scanning microscopy for both cell types and at various controlled conditions. Upon exposure to Cy5-cRGD-siRNA (important note: no transfection reagent was used to facilitate cellular uptake), most HUVEC cells produced strong fluorescent signal in cytoplasm (Figure 1B, upper left panel), while HeLa cells did not (Figure 1B, upper right panel). Uptake of fluorescently labeled control cRAD-siRNA by HUVEC cells was also negligent (Figure 1B, upper middle panel). These data demonstrate the ability of cRGD to effectively and specifically transport conjugated siRNAs inside the cells, expressing integrin αvβ3. Specificity of the uptake by HUVEC cells was confirmed and elaborated further, demonstrating that blocking of integrin αvβ3 on the cell surface by pre-incubation with either αvβ3-specific antibodies (Figure 1B, lower left panel) or un-conjugated cRGD (Figure 1B, lower middle panel) strongly interfered with Cy5-cRGD-siRNA uptake. At the same time, pre-incubation of HUVEC with un-conjugated control cRAD peptide did not affect the ability of Cy5-cRGD-siRNA to enter the cells (Figure 1B, lower right panel).

Due to structural and synthesis-related constraints, fluorescent peptide-siRNA conjugates described above had to be made with peptide moiety attached to the 5′-end of passenger (sense) strands and Cy5 fluorophore linked to the 5′-end of guide (antisense) strands of molecules. It has been demonstrated before, however, that the presence of phosphate at the 5′-end of a guide strand is critical for siRNA's ability to activate RNA-induced silencing complex (RISC) (24). Consistent with this, both unassisted uptake of Cy5-cRGD-siRNA and delivery of the molecule using conventional transfection reagent (Lipofectamine 2000) did not result in down-regulation of target mRNA (data not shown). To ensure RISC-activating capabilities, in the following experiments the test molecules contained phosphate, rather than Cy5, at the 5′-end of guide strands.

**Figure 2.**
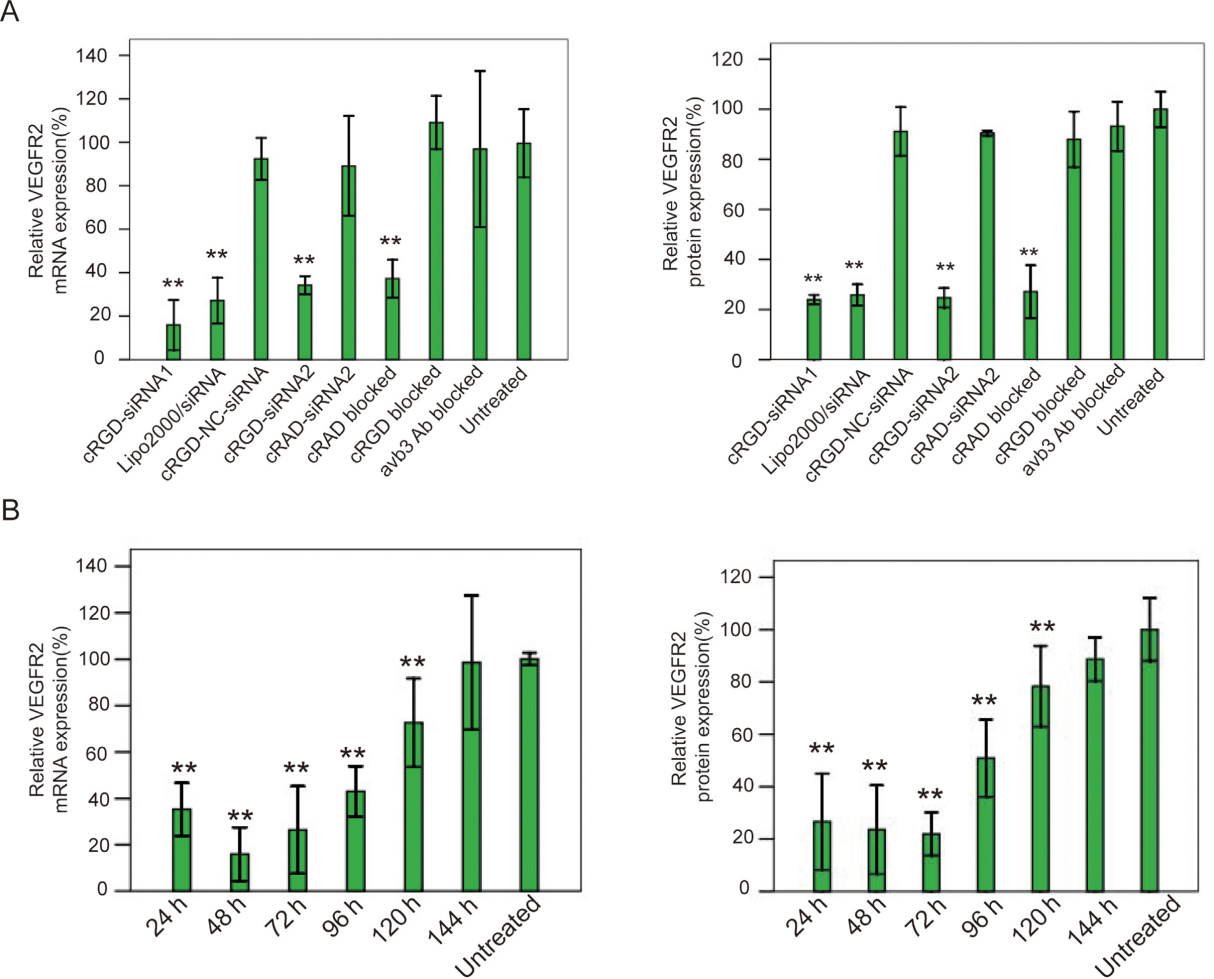
Efficacy and specificity of VEGFR2 gene silencing with cRGD-siRNA molecules *in vitro*. (**A**) Human αvβ3-positive HUVEC cells were treated (note: no transfection reagent was used) with two cRGD-siRNA conjugate molecules targeting different sites on VEGFR2 (cRGD-siRNA1 and cRGD-siRNA2), cRGD conjugate with non-targeting control siRNA (cRGD-NC-siRNA), VEGFR2 targeting siRNA conjugated to a control cRAD peptide (cRAD-siRNA2), or cRGD peptide alone (cRGD) (note: none of the molecules used for treatment was labeled with Cy5). For additional specificity tests, cells incubated with VEGFR2 targeting cRGD-siRNA2 were pre-treated with αvβ3-blocking antibodies (αvβ3 Ab blocked), cRGD peptide (cRGD blocked) or cRAD peptide (cRAD blocked). Treatment with conventional non-conjugated VEGFR2 siRNA using transfection reagent (Lipo2000/siRNA) was used as a knockdown positive control. Each treatment was done with molecules at 100 nM final concentration. After 12 h of incubation, cells were collected for mRNA and protein isolation. VEGFR2 mRNA (left panel) was quantified using RT-qPCR and normalized to internal control. Data are plotted relative to untreated cells (Untreated). VEGFR2 protein expression (right panel) was monitored and quantified using western blotting (the gel is not shown). The internally normalized data are plotted relative to untreated cells (Untreated). (**B**) The onset and longevity of gene knockdown were monitored in HEVEC cells at various time points after treatment with VEGFR2 cRGD-siRNA2. The time curves are shown for both VEGFR2 mRNA (left panel) and protein (right panel) expression levels. mRNA detection was conducted using internally normalized RT-qPCR. Protein expression was measured using internally normalized western blotting (the gel is not shown). Results are presented as means and standard deviations from triplicate data points. One (*) and two (**) stars indicate, correspondingly, *P* < 0.05 and *P* < 0.01 (versus untreated control groups).

Similarly to the experiments conducted with fluorescently labeled peptide-siRNA conjugates (described above), the ability of unlabeled, but containing phosphate at the 5′-end of the guide strand cRGD-siRNAs, to down-regulate gene expression was tested using unassisted cellular delivery, i.e. in the absence of a transfection reagent. To this end, the integrin αvβ3 positive HUVECs were treated with two different VEGFR2 targeting cRGD-conjugated siRNAs (cRGD-siRNA1 and cRGD-siRNA2; 100 nM each), and the intracellular levels of corresponding mRNA and protein were measured after 12 h of incubation (Figure 2A). Cells not treated with siRNA (Untreated) or treated with conjugated molecules containing non-targeting control sequence (cRGD-NC-siRNA) were used as negative controls. As a positive control, cells were transfected with 100 nM of conventional, not conjugated to cRGD VEGFR2-targeting siRNA, using Lipofectamine 2000 transfection reagent (Lipo2000/siRNA). qPCR analysis demonstrated that unassisted delivery of cRGD-siRNA1, cRGD-siRNA2 and the delivery of targeting siRNA using transfection reagent resulted in comparable mRNA down-regulation (about 80% for cRGD-siRNA1 and 65% for cRGD-siRNA2 versus 75% for siRNA/Lipofectamine 2000). On the protein level, cRGD-siRNA1 yielded 80%, and cRGD-siRNA2 produced 75% efficient knockdown of VEGFR2 versus 70% down-regulation achieved with siRNA/Lipofectamine 2000. The ability of cRGD-siRNA conjugates to knockdown genes was confirmed in an independent experiment with molecules, in which siRNA moieties were designed against a different target (GAPDH). These molecules also produced efficient gene down-regulation, both on the mRNA (∼75%) and the protein (∼60%) levels (Supplementary Figures S3A).

Specificity of knockdown using cRGD-conjugated siRNA molecules was confirmed using the approach described above for the fluorescently labeled molecules. Prior to the incubation with cRGD-siRNA conjugate (cRGD-siRNA2 in this case), HUVEC cells were first treated with αvβ3-blocking antibodies, cRGD peptide, or cRAD (control) peptide. As anticipated, pre-treatment with αvβ3 antibodies (αvβ3 Ab blocked) or cRGD peptide (cRGD blocked) inactivated αvβ3 integrin on the HUVECs surface, preventing uptake of functional cRGD-siRNA conjugates and, thus, abrogating target gene knockdown both on mRNA and protein levels (Figure 2A). Pre-treatment with cRAD peptide (cRAD blocked) did not have a negative effect on cRGD-siRNA knockdown efficiency. Additional support for the specificity of knockdown with cRGD-siRNA conjugates came from the demonstration that the same molecules that were functional in HUVEC (αvβ3-positive) cells failed to produce the knockdown in HeLa cells lacking αvβ3 integrin (Supplementary Figure S3B).

To test the dynamics and longevity of gene knockdown with cRGD-siRNA conjugates, HUVEC cells were treated with 100 nM of VEGFR2 cRGD-siRNA1, and the levels of target mRNA were measured at multiple time points after the treatment. The resulting time curve (Figure 2B) demonstrates that maximum knockdowns of mRNA and protein occur 48 and 72 h, correspondingly, after the exposure of cells to cRGD-siRNA, and is sustained (for both mRNA and protein) at statistically distinguishable level for up to 120 h after the initial treatment.

Taken together, these data demonstrate that cRGD-siRNA conjugates could specifically enter cells expressing integrin αvβ3 receptor without assistance of a transfection reagent (through unassisted uptake). Upon entry inside the cells, cRGD-siRNAs can activate RNAi machinery and produce efficient, specific and sustainable silencing of targeted genes.

### Injection of VEGFR2 cRGD-siRNA negatively affects angiogenesis in zebrafish embryos

Zebrafish lines expressing fluorescent protein markers under control of endothelium-specific promoters proved to become powerful animal models for investigating processes of blood vessel formation (including angiogenesis) and factors (including various dugs) affecting them (25–28). For our zebrafish *in vivo* studies, we have selected the well characterized Tg(*Flk:EGFP*) line expressing epidermal growth factor receptor (EGFP) fluorescent protein under control of FLK1 (also known as CD309, KDR or VEGFR2 (vascular endothelial growth factor receptor2, KDR/Flk-1)) promoter.

**Figure 3.**
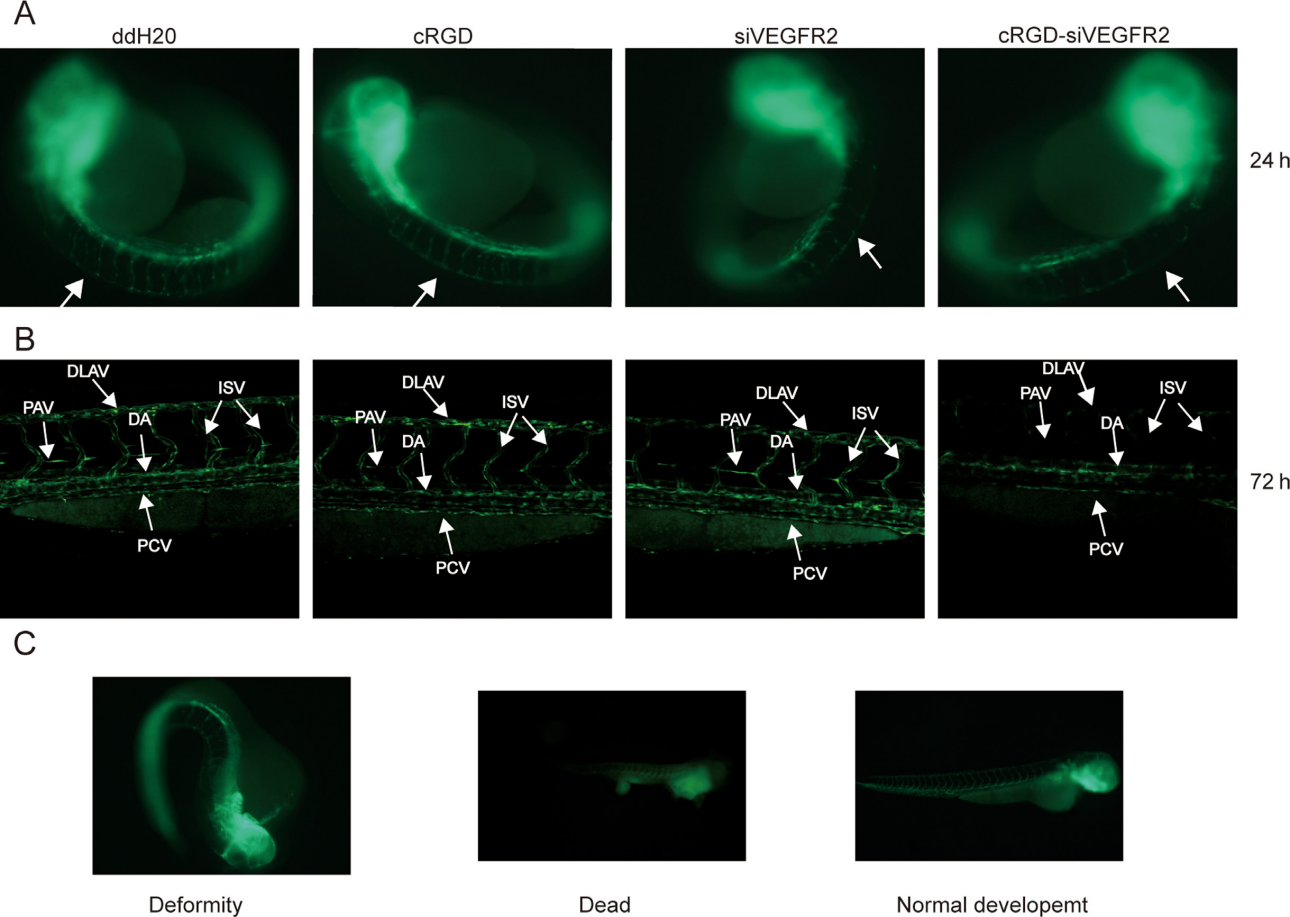
Anti-angiogenic effect of cRGD-siRNA in zebrafish. (**A**) The 5 hpf *Tg(flk1:EGFP)* zebrafish embryos were injected with either water (ddH20), cRGD peptide alone (cRGD), non-conjugated VEGFR2 targeting siRNA (siVEGFR2) or VEGFR2 cRGD-siRNA. Twenty-four hours after microinjection, angiogenesis in the whole embryos was visualized and imaged using Stereo Fluorescence Microscope. Arrows indicate the places of possible inhibition of trunk vascular development in animal treated with cRGD-siRNA (right panel) and corresponding places in control animals. (**B**) Animals treated as above were visualized for vasculature development using confocal microscopy 72 h after microinjections. Arrows indicate defective trunk vessel formation in animals treated with cRGD-siVEGFR2 (right panel) versus control group animals (three left panels): the barely visible inter-segmental vessels (ISV), dorsal longitudinal anastomotic vessels (DLAV), posterior cardinal vein (PCV) and parachordal vessels (PAV), as well as failure of a fraction of ISVs to branch off from the dorsal aorta (DA). (**C**) After injection of the 5 hpf zebrafish embryos with cRGD-siVEGFR2, during the 72 h observation period, majority of the animals developed into larvae normally (exemplified on the right panel). However, 15% of the animals developed abnormal phenotypes (exemplified on the left panel) and 10% of the animals died (exemplified on the middle panel).

Zebrafish embryos have been demonstrated to start expressing integrin αvβ3 by 48 hpf, but not by 24 hpf (29). The major vessels, including the dorsal aorta and the ventral veins, are fully developed by 24 hpf, followed by formation of angiogenic vessels, including the intersegmental vessels (ISV), between 24 and 72 hpf (30). To study the effect of VEGFR2 targeting cRGD-siRNA, we injected the molecule into 5 hpf zebrafish embryos and monitored phenotypes and patterns of blood vessel formation 24 and 72 h after injection. Non-conjugated siRNA, cRGD peptide alone or water were used for control treatments. Stereo fluorescence microscopy revealed no substantial difference in blood vessel development between experimental and control animals 24 h after treatment, except possible minor localized inhibition of trunk vasculature development in animals treated with cRGD-siRNA (Figure 3A). However, confocal microscopy 72 h after the injections demonstrated noticeable irregularities in animals treated with cRGD-siRNA, but not in the control group animals (Figure 3B). These included hardly visible inter-segmental vessels (ISV), dorsal longitudinal anastomotic vessels (DLAV), posterior cardinal vein (PCV) and parachordal vessels (PAV), as well as the failure of a fraction of ISVs to branch off from the dorsal aorta (DA). During the course of the observation (24–72 h after the injection into 5 hpf embryos), the majority of zebrafish animals developed normally from embryos within chorioallantoic membrane into larvae. However, 15% of animals treated with cRGD-siRNA were found to develop visually obvious deformities, and 10% of the animals died (Figure 3C).

Taken together, these data indicate that cRGD-conjugated siRNA molecule targeting VEGFR2 is active in zebrafish embryos after the injection, and can generate phenotypes consistent with the role of its target in animal's vasculature formation.

### Bio-distribution and *in vivo* gene knockdown with cRGD-siRNA in mouse tumors

**Figure 4.**
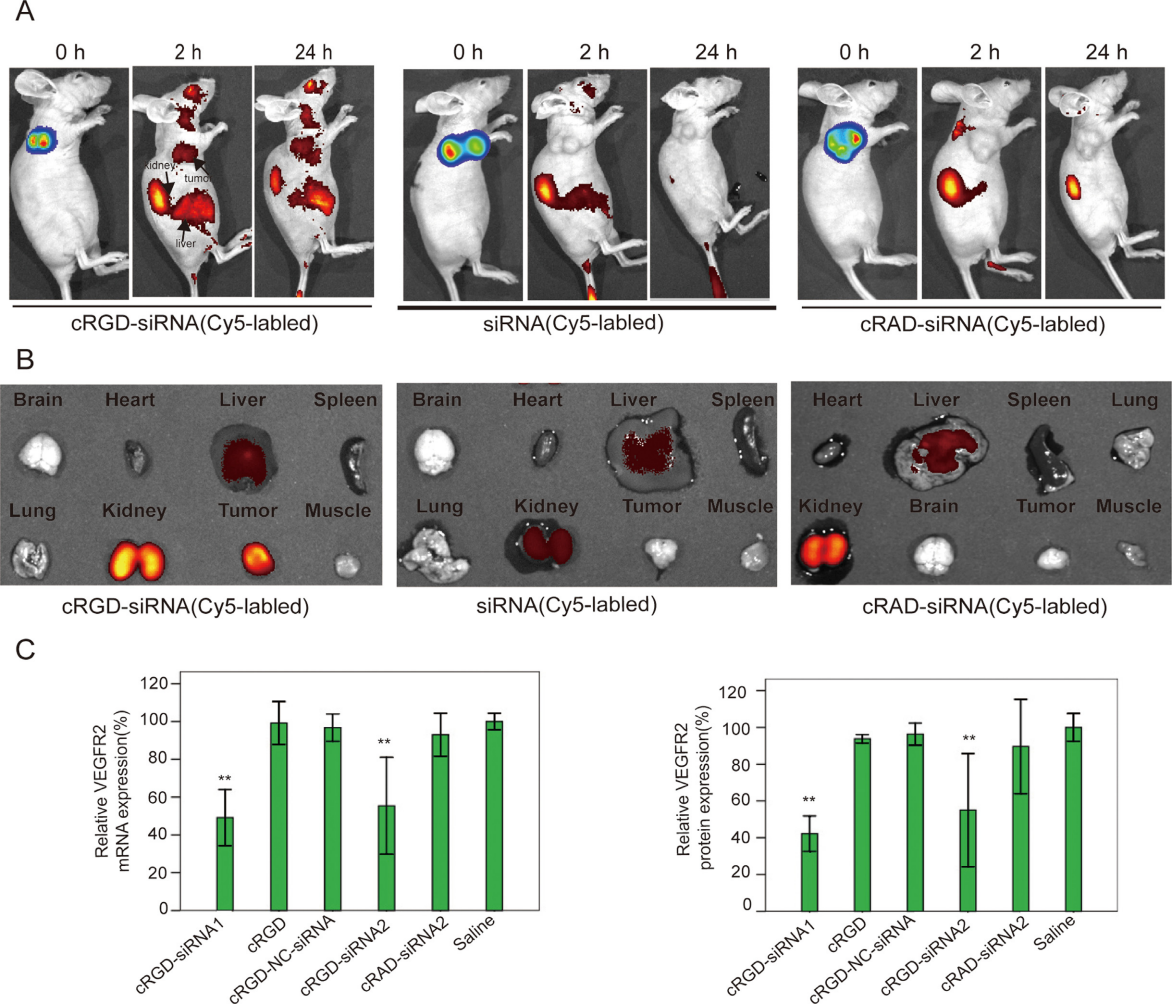
*In vivo* cRGD-siRNA bio-distribution and gene silencing. (**A**) BALB/c nude mice from three groups were first injected subcutaneously (right shoulder area) with A549-luc cells and allowed to develop tumors, which are visible physically and can be evaluated quantitatively (in luciferase emission spectrum) using Xenogen IVIS *In Vivo* Imaging System (0 h time point). At this point animals were injected (tail vein, single dose, 1 nmol/dose) with cRGD-conjugated siRNA, non-conjugated siRNA or siRNA conjugated with control cRAD peptide (siRNAs in each case were labeled with Cy5). The whole animal images were then taken using Xenogen IVIS *In Vivo* Imaging (in Cy5 emission spectrum) at 2 and 24 h time points. (**B**) Individual organs were isolated from animals 24 h after injection with Cy5-labeled cRGD-siRNA (left panel), non-conjugated siRNA (middle panel) or cRAD-siRNA (right panel) and bioimaged using the IVIS imaging system (in Cy5 emission spectrum). (**C**) Knockdown of VEGFR2 mRNA and protein. Animals bearing A549-luc tumors were injected with either one of two cRGD-conjugated siRNAs (cRGD-siRNA1 and cRGD-siRNA2), or controls (cRGD alone, cRGD conjugated to non-targeting control siRNA or siRNA conjugated to a control cRAD peptide) 6× every 3 days. mRNA samples were prepared from tumors 2 days after the last treatment and analyzed using qPCR. VEGFR2 expression was normalized to GAPDH mRNA. Protein samples were prepared from tumors described in left panel of (C), and visualized (gel not shown) and quantified using western blotting. Data points represent the means and standard deviations from triplicate samples. Single stars (*) denote *P* < 0.05, and double stars (**) stand for *P* < 0.01 versus untreated control groups.

Bio-distribution of chemically stabilized cRGD-siRNA in tumor-bearing mice was examined by whole animal bioluminescence analysis using IVIS (Xenogen) Spectrum *In Vivo* Imaging system. Nude mice have developed visualizable tumors in the right shoulder area after subcutaneous injection into that place of human luciferase-expressing A549-luc cells. Tumors were imaged in luciferase emission spectrum after reaching volumes of 40–50 mm^3^ (Figure 4A; 0 h time points). Subsequently, animals were injected with 1 nmol (∼0.753 mg/kg) of Cy5-labeled cRGD-siRNA or control molecules. Bioimaging in Cy5 emission spectrum (Figure 4A; 2 and 24 h time points) revealed a strong fluorescent signal in the tumors of mice injected with cRGD-siRNA (Figure 4A; left panel), while no signal was detected in mice injected with chemically stabilized non-conjugated siRNA (Figure 4A; middle panel), or siRNA conjugated with control cRAD peptide (Figure 4A; right panel). Fluorescent signals in tumors of mice injected with cRGD-siRNA were detected as early as 30 min after the injection (not shown), and lasted for at least 24 h (Figure 4A; 24 h time points). In mice injected with Cy5-cRGD-siRNA, in addition to tumors, substantial fluorescence was detected in kidneys and liver, indicating biodistribution to these organs, but also suggesting renal and/or hepatobiliary routes for molecule clearance (31). Twenty-four hours after the injection, mice injected with Cy5-labeled siRNAs were euthanized, tumors and individual organs excised and prepped for analysis using IVIS (Xenogen) imaging system (Figure 4B). *Ex vivo* bioimaging in Cy5 emission spectrum confirmed the initial whole animal imaging observations, in particular the significantly higher fluorescent signal coming from the tumors of mice injected with cRGD-conjugated siRNA (left panel) versus fluorescence coming from the tumors of mice injected with non-conjugated molecule (middle panel) or siRNA conjugated with control cRAD peptide (right panel). In additional experiment using Cy5-labeled siRNA, tumor-bearing animals were injected consecutively every 3 days, with six total number of injections. Two days after the last injection the whole animal and tumor/organ imaging was conducted, demonstrating strong fluorescence signal coming predominantly from tumors of animals injected with cRGD-conjugated molecules (Supplementary Figure S4A).

As discussed above, Cy5-labeled peptide-conjugated siRNA molecules, while convenient for *in vitro* localization and *in vivo* biodistribution bioimaging, cannot be used for the gene knockdown studies. Indeed, due to structural limitations, the Cy5 fluorophore in test molecules was attached to the 5′-end of siRNA guide strand, replacing phosphate group required for the activation of RNAi machinery. Therefore, the following functional studies of cRGD-siRNA conjugates were conducted with molecules containing phosphate, but lacking Cy5 fluorescent label at the 5′-end of siRNA's guide strands. To this end, BALB/c nude mice with developed A549-luc tumors were injected intravenously with one of the two cRGD-conjugated siRNAs (cRGD-siRNA1 and cRGD-siRNA2), or one of the controls (cRGD alone, cRGD conjugated to non-targeting control siRNA or siRNA conjugated to a control cRAD peptide) 6× every 3 days. Analysis of the excised tumor samples 2 days after the last injection (day 17) revealed >50% knockdown of targeted VEGF2 mRNA (as determined by RT-qPCR) with both cRGD-siRNA1 and cRGD-siRNA2 (Figure 4C, left panel). Quantitative western blotting analysis demonstrated almost 70% reduction of corresponding protein with cRGD-siRNA1 and >50% protein reduction with cRGD-siRNA2 (Figure 4C, right panel). In similarly conducted experiments, a cRGD-conjugated siRNA designed against a different gene, β-actin, also bio-distributed and accumulated in tumors and produced nearly 40% down-regulation of targeted mRNA and corresponding protein (Supplementary Figures S4B and 4C).

Taken together, these data demonstrate that in mouse model system siRNAs conjugated to targeting cRGD peptide bio-distribute to tumors and down-regulate specifically and efficiently expression of targeted genes.

### Inhibition of tumor growth and angiogenesis using VEGFR2 cRGD-siRNA in mouse model system

To test the potential of cRGD-navigated siRNAs as anticancer agents, we investigated the effect of systemic injection of conjugate molecules targeting VEGFR2 on the tumor development and angiogenesis in mouse model system. For this, the BALB/c nude mice with developed A549-luc (luciferase expressing) tumors were injected with either one of the two VEGFR2 targeting cRGD-siRNAs (cRGD-siRNA1 or cRGD-siRNA2), or one of the following controls: cRGD peptide alone (cRGD), cRGD conjugated to a control non-targeting siRNA molecule (cRGD-control siRNA), siRNA targeting VEGFR2 conjugated to a control cRAD peptide (cRAD-siRNA2), cRGD conjugated to siRNA targeting non-essential for tumor growth β actin gene (cRGD-β actin siRNA), saline injection solution (saline). Six consecutive injections were conducted every 3 days, and the progression of tumors was visualized by bio-imaging and quantitatively measured at various time points.

**Figure 5.**
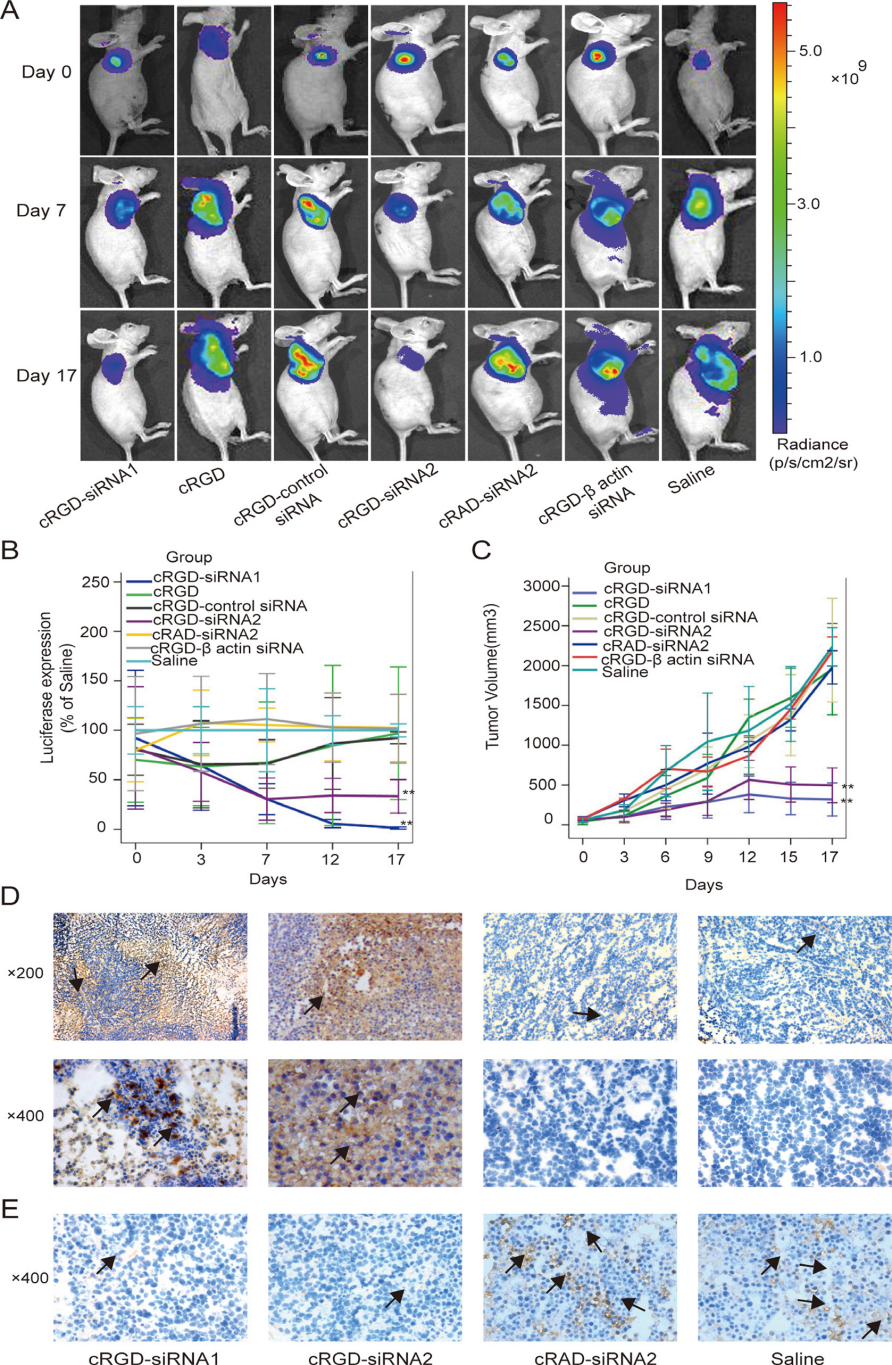
Effect of systemic delivery of VEGFR2 cRGD-siRNAs on tumor growth and angiogenesis in the mouse model system. Seven groups of BALB/c nude mice with developed A549-luc (luciferase expressing) tumors were tail vein injected 6× every 3 days with 1 nmol solutions of either siRNA targeting VEGFR2 conjugated to cRGD (cRGD-siRNA1), cRGD peptide alone (cRGD), cRGD conjugated to non-targeting control siRNA (cRGD-control siRNA), another siRNA targeting VEGFR2 conjugated to cRGD (cRGD-siRNA2), VEGFR2 targeting siRNA conjugated to control cRAD peptide (cRAD-siRNA2), cRGD conjugated to siRNA targeting unrelated to tumor growth and angiogenesis β-actin gene (cRGD-β actin siRNA) or saline solution alone (saline). (**A**) Animals from each test group were bioimaged (Xenogen IVIS system) at indicated time points in luciferase emission spectrum to visualize the progression of tumor growth. (**B**) For each group of animals the intensity of luciferase signal coming from engrafted tumors was quantitatively measured (Xenogen IVIS system) on indicated days of treatment and plotted (normalized to data from saline treated animals). (**C**) Sizes of engrafted tumors were measured for each group of animals on each of the injection day (every 3 days), as well as 2 days after the last injection, and the corresponding tumor growth curves were generated. (**D**) Tumor tissues from animals from 4 (cRGD-siRNA1; cRGD-siRNA2; cRAD-siRNA2 and saline) out of 7 initial treatment groups were isolated on day 17 (from first injection) and analyzed for apoptosis using TUNEL staining (arrows indicate apoptotic cells). (**E**) Immunohistochemical staining for CD31 was used to analyze density of vascularization in the same tumor tissue samples as in (D) (arrows indicate signals originating from CD31 antibodies). For quantitative measurements experiments were performed in triplicates. Double stars (**) denote *P* < 0.01.

Bio-imaging using Xenogen IVIS system revealed a progressively lower signal coming from mice treated with functional cRGD-siRNA molecules versus control treated animals (Figure 5A). Such visual observation was confirmed when luciferase signals were quantified and normalized, with the lowest intensities recorded for animals treated with VEGFR2 targeting cRGD-siRNAs on day 17 after the initial injection (Figure 5B). Physical (size) measurements of tumors demonstrated significantly reduced, almost stalled, growth rates (*P* < 0.01) after the treatment with VEGFR2 cRGD-siRNAs, but not with any of the controls (Figure 5C).

Inhibition of tumor angiogenesis reduces supply of nutrition from blood to cancer cells, which may cause their apoptosis and overall negatively affect tumor growth. Visualization of tissue samples (on day 17 after the first injection) using TUNEL assay demonstrated a significantly higher number of apoptotic cells in tumors from VEGFR2 cRGD-siRNA1 or cRGD-siRNA2 treated mice, when compared to the tumor tissues prepared from the control VEGFR2 cRAD-siRNA2 or saline treated animals (Figure 5D). Immunohistochemical analysis using CD31 staining (which detects endothelial cells of expanding vasculature) also revealed a substantially lower presence of micro-vessels in tumors from VEGFR2 cRGD-siRNA1 or cRGD-siRNA2 versus VEGFR2 cRAD-siRNA2 or saline treated animals (Figure 5E). Taken together, the data argue that the injection of VEGFR2 targeting cRGD-siRNA conjugate molecules results in the effective and specific reduction of tumor growth, likely due to the inhibition of tissue angiogenesis and apoptosis of cancer cells.

It has been reported that unmodified siRNAs can induce innate immune responses, as well as sequence-independent antiangiogenic activity (32,33). To alleviate such potential non-specific effects, we introduced chemical modifications 2′-O-Me moieties for riboses of three nucleotides on each end of both siRNA strands. To test the possible immunostimulatory effects of cRGD-siRNA conjugates used in the study, serum levels of cytokines were measured in nude mice after the single injection of cRGD-siRNA1. No significant difference has been detected in the levels of interferons α and γ, as well as interleukins 6 and 12, between mice treated with the test cRGD-siRNA1, control cRGD peptide or saline 6 and 24 h after the injection (Supplementary Table S3 and Supplementary Figure S5A).

To test the overall toxicity of cRGD-siRNA conjugates *in vivo*, we monitored the creatinine levels (Cr; indicator of renal health) and alanine transferase activity (ALT; indicator of liver damage) in mice treated with cRGD-siRNA1 (injected 6× every 3 days). No statistically meaningful difference has been detected between mice treated with test molecule or control saline 2 days after the last injection (Supplementary Tables S4 and Figure S5B), indicating no renal and liver toxicity. In addition, no morbidity or weight loss has been detected in mice, following the 16-day treatment with cRGD-siRNA1 and the corresponding controls (data not shown). Taken together, the data argue for the potent and specific negative effect of VEGFR2 cRGD-siRNA molecule on tumor angiogenesis and growth after systemic delivery. At the same time, the active molecules demonstrate no general toxicity after prolonged and extensive treatment.

## DISCUSSION

RNAi technology offers immense therapeutic promise due to high potency, specificity and universality of its siRNA compounds. To utilize this promise, the challenge of siRNA *in vivo* delivery to the disease causing tissues and cell types has to be addressed. The majority of current approaches for systemic delivery of unmodified siRNAs recruit lipid nano-particles (LNPs)—to provide stability, organ/tissue biodistribution and cellular uptake (34). There are several challenges with the use of LNPs, however, which are difficult to address simultaneously. Due to their large size, LNPs can be introduced into blood circulation only through intravenous administration route (35). For the same reason, these particles biodistribute predominantly to liver and, to a lesser degree, to spleen and kidney. These are the very few organs containing fenestrae, or endothelial structures allowing large particles to cross from blood into the organ's interstitium (36). In addition, unmodified siRNAs (frequently used in LNP formulations) can cause non-specific immunostimulatory effects, and the LNP particles themselves often stimulate general toxicity (33,37). Finally, the LNP-siRNA complexes are difficult and expensive to manufacture, which may become a serious hurdle when developing therapeutic drugs.

To address the challenges associated with the use of LNPs, several developers of siRNA therapeutics turned to chemical modification and conjugation strategies. Chemical modifications can dramatically increase nuclease protection and diminish immunostimulatory properties of siRNAs (38), while various conjugated moieties may allow wider selection of targeted organs (39). Chemically modified siRNA conjugates are relatively small in size, which also allows using more patient-friendly (versus intravenous) subcutaneous administration route for systemic drug delivery.

Currently, the most advanced therapeutic program engaging RNAi chemical modification and conjugation strategy is Alnylam's Phase II ALN-TTRsc (USA clinical trial NCT01981837; http://clinicaltrials.gov/show/NCT01981837). The program utilizes a chemically modified siRNA molecule conjugated to *N*-acetylgalactosamine (GalNAc) moiety, which targets it specifically to the asialoglycoprotein receptors (ASGPR), highly expressed by liver hepatocytes. In liver, the Alnylam's GalNAc siRNA conjugates are aimed to silence expression of transthyretin (TTR) protein, a mutant form of which causes TTR-mediated amyloidosis (ATTR), an inherited, progressively debilitating, frequently fatal disease (Manoharan M. *et al.*, personal communication).

This study used similar principles to develop siRNA molecules targeted to cancer tumors, rather than to the liver. Solid tumors typically demonstrate enhanced angiogenesis and develop expanding vasculature, which is characterized by high expression levels of integrins, cell surface receptors that promote endothelial cell migration and survival. cRGD peptide is a strong and specific ligand for αvβ3 integrin, selectively over-expressed on endothelial cells of expanding (but not quiescent) blood vessels, and found in lung and breast cancers (40,41), melanomas (42) and brain tumors (43). Earlier *in vitro* studies demonstrated the ability of cRGD-siRNA conjugates to effectively and specifically enter cells expressing αvβ3 integrin and to down-regulate the expression of a selected gene (16). We first confirmed such findings by showing that cRGD peptide can specifically direct conjugated siRNA molecules inside the αvβ3-expressing cells (Figure 1), resulting in the effective knockdown of selected genes (Figure 2). We then demonstrated that cRGD-siRNA conjugates biodistribute in mice to the engrafted tumors after systemic intravenous delivery *in vivo*. Delivery of the cRGD-siRNAs to the tumors resulted in the specific and effective down-regulation of targeted genes (Figure 4). Finally, we showed that down-regulation of VEGFR2 (one of the genes known to be required for angiogenesis) in mouse tumors resulted in reduced blood vessel formation and substantially slower tumor growth (Figure 5). Consistent with findings in mice, down-regulation of VEGFR2 in developing zebrafish embryos negatively affected development of the blood vessel system (Figure 3).

Taken together, the obtained results provide a strong proof-of-concept for the potential use of cRGD conjugation strategy in targeting siRNA molecules to solid tumors and, in particular, for the use of VEGFR2 cRGD-siRNA as anticancer therapeutics. Additional studies, however, will need to be conducted to take this potential to the next level of drug development, and might include: (i) medicinal chemistry studies of the molecules to optimize ligand moiety's performance (e.g. earlier studies demonstrated that the use of multiple cRGDs may enhance the affinity; (16)) and stability of the siRNA component (although basic 2′-O-Me chemical modifications were used in this study to enhance siRNA nuclease resistance, more diverse and complex chemical modification patterns may further improve stability of the molecule; Manoharan M *et al.*, personal communication); (ii) toxicology and safety animal studies (although our initial tox and immunostimulatory tests produced encouraging results, more detailed studies, also involving non-human primates, will need to be conducted).

## SUPPLEMENTARY DATA

Supplementary Data are available at NAR Online.

SUPPLEMENTARY DATA
