# Supplementary material for: Tumor-targeted in vivo gene silencing via systemic delivery of cRGD-conjugated siRNA
Source: Nucleic Acids Res. 2014 Sep 15;42(18):11805–17. doi: 10.1093/nar/gku831 (PMC4191406; doi:10.1093/nar/gku831)
Supplement: SUPPLEMENTARY DATA [file supp_42_18_11805__index.html]

Tumor-targeted in vivo gene silencing via systemic delivery of cRGD-conjugated siRNA — SUPPLEMENTARY DATA 

# Tumor-targeted *in vivo* gene silencing *via* systemic delivery of cRGD-conjugated siRNA

## SUPPLEMENTARY DATA

**Files in this Data Supplement:**

- SUPPLEMENTARY DATA
