# Supplementary material for: Tumor-targeted in vivo gene silencing via systemic delivery of cRGD-conjugated siRNA
Source: Nucleic Acids Res. 2014 Sep 15;42(18):11805–17. doi: 10.1093/nar/gku831 (PMC4191406; doi:10.1093/nar/gku831)
Supplement: SUPPLEMENTARY DATA [file supp_gku831_nar-02041-y-2014-File007.pdf]

Supplementary Figure S1

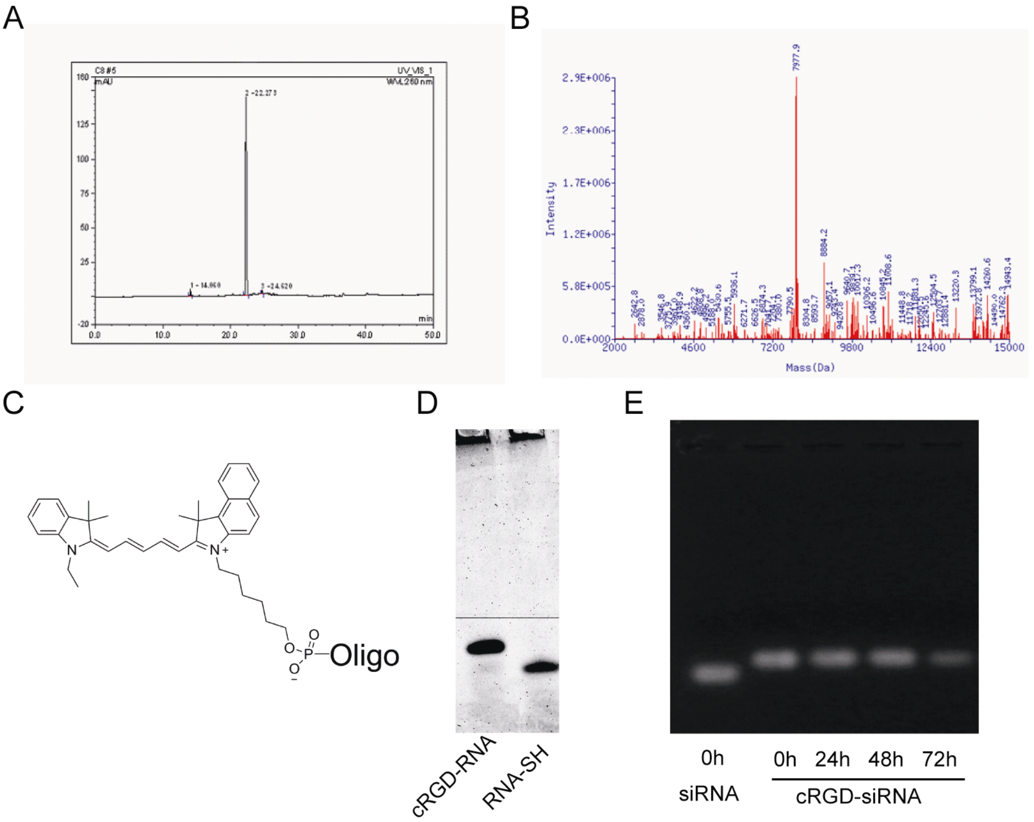

Supplementary Figure S1. Purity, structure and nuclease resistance of synthetic peptide-siRNA conjugates. (**A**) An exemplary RP-HPLC analytic chromatogram of a purified cRGD-conjugated single-stranded RNA (cRGD-ssRNA, where ssRNA component is the siRNA passenger strand) demonstrates >90% purity of synthetic molecules. (**B**) An exemplary mass-spectrometric analysis of purified cRGD-ssRNA (mouse VEGFR2-siRNA-1 in **Table S2**) demonstrates close proximity of experimentally detected (7977.9 Da) and theoretically predicted (7975.3 Da) molecular weight values. (**C**) Chemical structure of the Cy5 5'-end label used in the study. (**D**) Denaturing urea-polyacrylamide (20%) gel electrophoresis analysis revealed a characteristic shift in molecular weight-dependent mobility between conjugated and non-conjugated molecules. (**E**) To test cRGD-siRNA resistance to blood nucleases, molecules were incubated in 100% mouse serum at 37°C at indicated time periods and then analyzed

using agarose (1.2%) gel electrophoresis.

Supplementary Figure S2

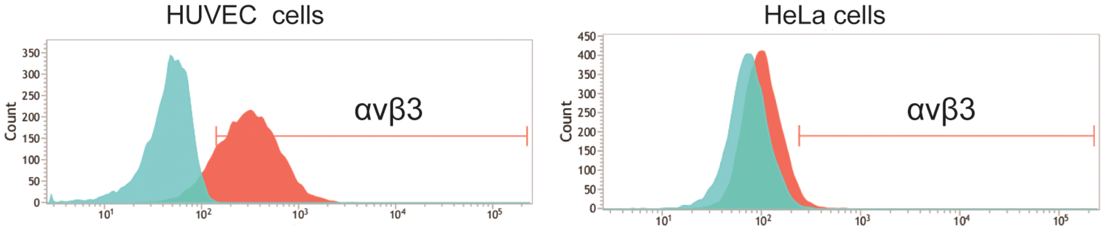

Supplementary Figure S2. Analysis of integrin αvβ3 expression on surface of human umbilical vein endothelial cells (HUVEC) *vs*. HeLa cells. HUVEC (left panel) and HeLa cells (right panel) were sorted with (red) or without (blue) αvβ3-specific antibodies using FACSCalibur (BD Biosciences) flow cytometer and analyzed using CellQuest 3.0 software. Characteristic peak shift in the presence of specific antibodies is more pronounced for HUVEC cells, supporting earlier findings that these, but not HeLa cells express αvβ3 integrin on the cell surface.

Supplementary Figure S3

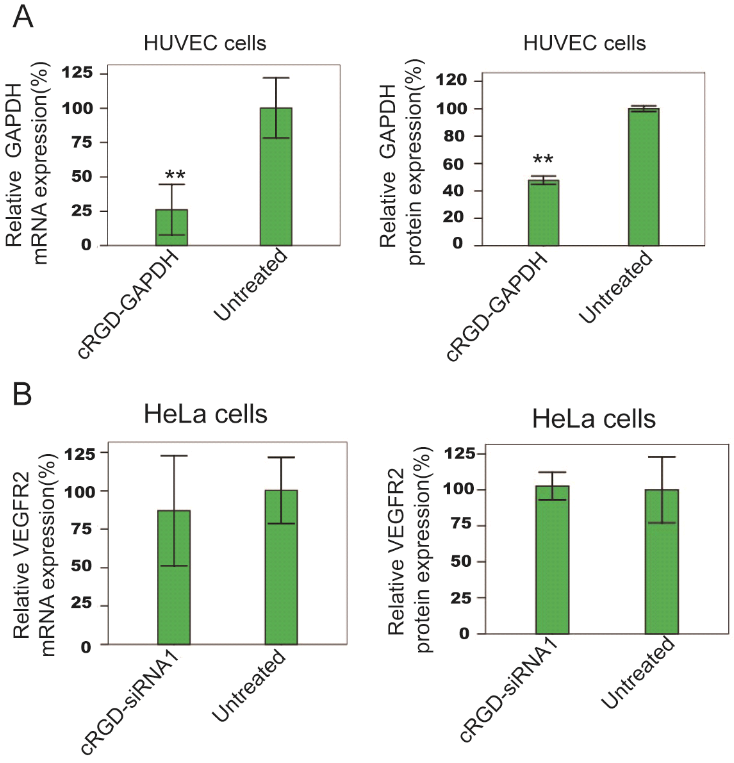

Supplementary Figure S3. Efficacy and specificity of cRGD-siRNA mediated gene silencing *in vitro.* **(A)** HUVEC (αvβ3-positive) cells were treated (note: no transfection reagent was used) with 100 nM cRGD-siRNA targeting GAPDH. Cells were collected after 24 hours of incubation and expression of mRNA (left panel) and protein (right panel) was assessed using RT-qPCR and Western blotting (gel is not shown), correspondingly. **(B)** HeLa (αvβ3-negative) cells were treated (note: no transfection reagent was used) with 100 nM cRGD-siRNA1, a conjugate molecule demonstrated to be effective in HUVEC (αvβ3-positive) cells (see Fig 2)**.** Data points represented the means and standard deviations from triplicates. Two (**) stars indicate P< 0.01 (*vs.* untreated control groups).

Supplementary Figure S4

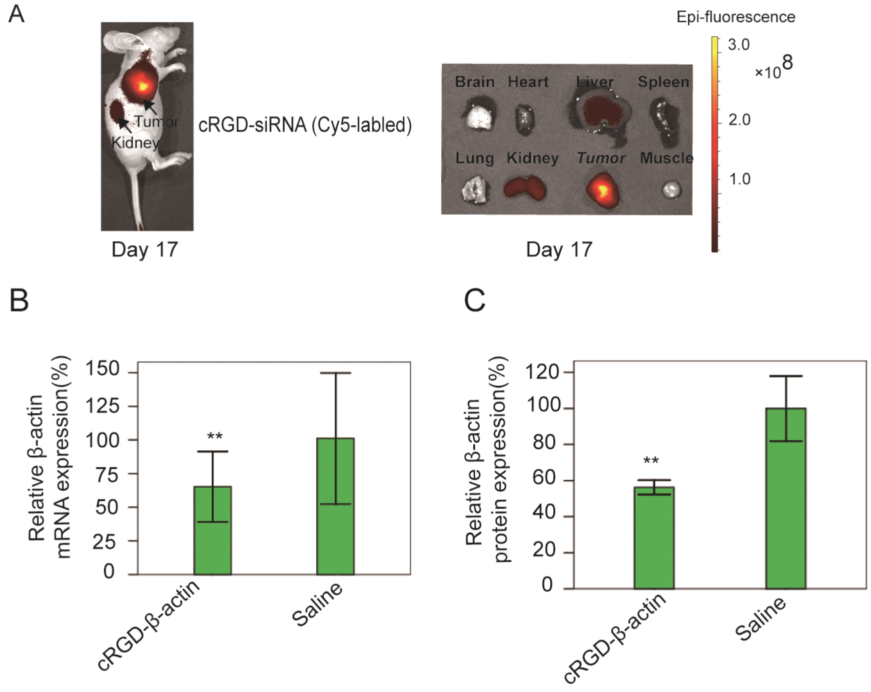

Supplementary Figure S4. Bio-distribution and knockdown with β-actin cRGD-siRNA. Experiments were conducted essentially the same way as described in Figure 4, except that the siRNA used in the study was designed to target β-actin, rather than VEGF2 gene. **(A)**

Tumor-bearing mice were injected with Cy5-labeled cRGD-siRNA intravenously six times, once every two days (1 nmol/injection), and the whole animals (left panel) or individually isolated organs/tumors (right panel) were bio-imaged two days after the last injections. Tumor samples were then analyzed for knockdown of β-actin mRNA using RT-qPCR (**B**) and of corresponding protein using quantitative Western blotting (**C**). All results were compiled from triplicate data sets. Double stars (**) denote the P<0.01 values.

Supplementary Figure S5

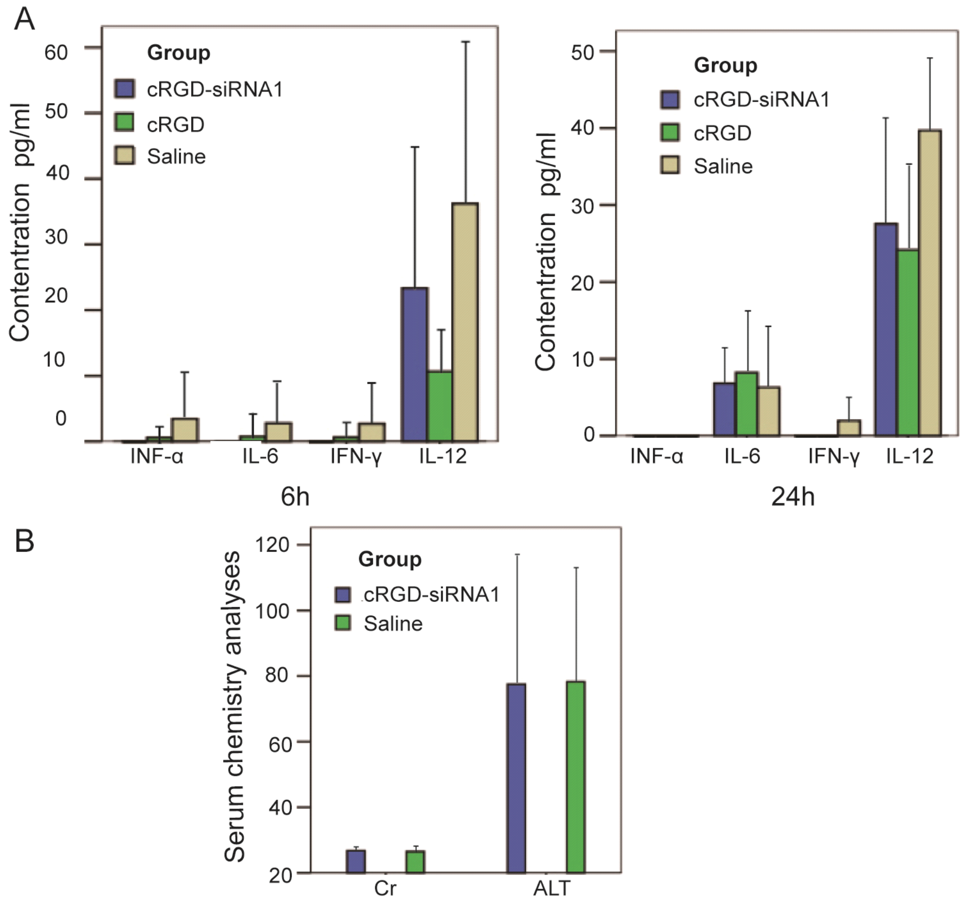

Supplementary Figure S5. Immuno-stimulatory and toxicity profiles of cRGD-siRNA conjugate molecules *in vivo*. (**A**) Athymic nude female mice were tail vein injected with VEGFR2 targeting cRGD-siRNA1, cRGD peptide alone or saline. Mouse serum was collected 6 hours and 24 hours after the injection, and cytokine levels (indicators of immunostimulatory

effects) were analyzed using ELISA. (**B**) Nude mice were injected intravenously with cRGD-siRNA1 (1 nmol/injection) or saline every three days for a total of six times. Serum was collected two days after the last injection and analyzed for the alanine aminotransferase activity (ALT; IU/L; indicator of liver toxicity) and creatinine levels (Cr; μmol/L; indicator of renal health). In all experiments three animals were used per each treatment.

Supplementary Table S1. Sequences of qRT-PCR primers used in the study

Name	Sequence
Mouse VEGFR2	Forward primer: 5'-AGAATGCGGGCTCCTGACTA-3'<br>Reverse primer: 3'-CCATGCTCAGTGTCTCTGACA-5'
Human VEGFR2	Forward primer: 5'-GCCAGTCTTCTAGGCATATCC-3'<br>Reverse primer : 3'-CTCCCCAGGTACTGCTACTT-5'
Mouse 18s rRNA	Forward primer : 5'-CCTGGATACCGCAGCTAGGA-3'<br>Reverse primer : 3'-GCGGCGCAATACGAATGCCCC-5'
Human GAPDH	Forward primer : 5'-AACGGATTTGGTCGTATTGG-3'<br>Reverse primer: 3'-GATCTCGCTCCTGGAAGATG-5'

Supplementary Table S2. Sequences and structures of cRGD-siRNAs used in the study

Name	The precise structures of the molecules	Target Masses	Observed Masses
cRGD-siRNA1<br>(Mouse)	Sense strand: 5'-mCmGmGAGAAGAAUGUGGUmUmAmAdTdT-3'-cRGD<br>Antisense strand: 3'-dTdTmGmCmCUCUUCUUACACCAmAmUmU-phos-5'	7977.4<br>6652.1	7977.9<br>6652.5
cRGD-siRNA1<br>(Cy5-labled, Mouse)	Sense strand: 5'-mCmGmGAGAAGAAUGUGGUmUmAmAdTdT-3'-cRGD<br>Antisense strand: 3'-dTdTmGmCmCUCUUCUUACACCAmAmUmU-Cy5-5'	7977.4<br>7165.7	7977.9<br>7165.7
cRGD-siRNA2<br>(Mouse)	Sense strand: 5'-mGmAmGUGUAAAAACAUUUmGmAmA dTdT-3'-cRGD<br>Antisense strand: 3'-dCdAmCmUmCACAUUUUUGUAAAmCmUmU-phos-5'	7906.3<br>6672.1	7906.1<br>6672.9

cRAD-siRNA2 (Mouse)	Sense strand: 5'-mGmAmGUGUAAAAACAUUmGmAmAdTdT-3'-cRAD<br>Antisense strand: 3'-dCdAmCmUmCACAUUUUUGUAAAmCmUmU-phos-5'	7920.3<br>6672.1	7920.5<br>6671.9
cRAD-siRNA2 (Cy5-labled, Mouse)	Sense strand: 5'-mGmAmGUGUAAAAACAUUmGmAmAdTdT-3'-cRAD<br>Antisense strand: 3'-dCdAmCmUmCACAUUUUUGUAAAmCmUmU-Cy5-5'	7991.5<br>7165.7	7991.5<br>7166.5
cRGD-siRNA1 (Human)	Sense strand: 5'-mGmGmUAAAGAUUGAUGAAmGmAmAdTdT-3'-cRGD<br>Antisense strand: 3'-dTdTmCmCmAUUUCUAACUACUUmCmUmU-phos-5'	7985.4<br>6614.0	7985.5<br>6613.6
cRGD-siRNA2 (Human)	Sense strand: 5'-mGmCmCACCAUGUUCUCUAmAmUmA dTdT-3'-cRAD<br>Antisense strand: 3'-dTdA mCmGmGUGGUACAAGAGAUmUmAmU-phos-5'	7745.1<br>6907.3	7745.6<br>6907.8
cRGD-siRNA (Zebrafish)	Sense strand: 5'-mCmUmGAAAACAAUGUUGUmGmAmA dTdT-3'-cRGD<br>Antisense strand: 3'-dTdTmGmA mCUUUUGUUACAACAmCmUmU-phos-5'	7882.3<br>6717.1	7882.4<br>6717.6

siRNA, small interfering RNA; "mN" indicates 2'-O-methyl ribose modification (2'-O-Me); "phos" stands for phosphorylated

Supplementary Table S3A. Cytokine levels in mouse sera 6 hours after treatment (n=3, mean±SD)

Cytokine	cRGD-siRNA1	cRGD	Saline	Unit
INF-α	0.00±0.00	0.57±0.66	3.46±3.02	pg/mL
IL-6	0.00±0.00	0.75±1.31	2.75±2.39	pg/mL
INF-γ	0.00±0.00	0.63±0.95	2.70±2.40	pg/mL
IL-12	23.36±7.83	10.67±1.94	36.20±9.28	pg/mL

Supplementary Table S3B. Cytokine levels in mouse sera 24 hours after treatment (n=3, mean±SD)

Cytokine	cRGD-siRNA1	cRGD	Saline	Unit
INF-α	0.00±0.00	0.00±0.00	0.00±0.00	pg/mL
IL-6	6.82±4.35	8.23±7.08	6.30±6.57	pg/mL
INF-γ	0.00±0.00	0.00±0.00	1.95±1.68	pg/mL

IL-12	27.54±5.37	24.17±3.92	39.68±2.80	pg/mL

Supplementary Table S4A. Creatinine (Cr) levels in mouse sera after treatment (n=3, mean±SD)

Treatment	# of animals	Mean±SD	Unit
Saline	3	26.50±1.29	μmol/L
cRGD-siRNA1	3	26.75±3.20	μmol/L
P		0.89	

Supplementary Table S4B. ALT levels in mouse sera after treatment (n=3, mean±SD)

Treatment	# of animals	Mean±SD	Unit
Saline	3	78.25±11.09	IU/L
cRGD-siRNA1	3	77.50±12.07	IU/L
P		0.93	
